# A brief intervention to reduce burnout and improve sleep quality in medical students

**DOI:** 10.1186/s12909-020-02263-6

**Published:** 2020-10-06

**Authors:** Jennifer R. Brubaker, Aili Swan, Elizabeth A. Beverly

**Affiliations:** 1 Department of Medicine, Ohio University Heritage College of Osteopathic Medicine, Athens, OH 45701 USA; 2 Department of Primary Care, Ohio University Heritage College of Osteopathic Medicine, Athens, OH 45701 USA; 3 The Diabetes Institute, Ohio University, Athens, OH 45701 USA; 4 Heritage Faculty Endowed Fellowship in Behavioral DiabetesOHF Ralph S. Licklider, D.O., Research Endowment, Athens, USA

**Keywords:** Burnout, Smartphone use, Osteopathic medical students, Intervention

## Abstract

**Background:**

Perceived stress, burnout, and poor sleep quality are high among medical students. Interventions designed to target these issues are necessary to promote the health and well-being of medical students. The purpose of this study was twofold: 1) to assess the feasibility of implementing a sunrise alarm clock intervention with medical students and 2) to evaluate the impact of the intervention on perceived stress, burnout scores, and sleep quality.

**Methods:**

We conducted a feasibility study to evaluate the efficacy of a two-week, sunrise alarm clock intervention in combination with electronic device removal at bedtime. We assessed first- and second-year medical students’ perceived stress, burnout scores, including Emotional Exhaustion, Depersonalization, and Low Sense of Personal Achievement, and sleep quality before and after the intervention. In addition, we measured smartphone addiction prior to the intervention.

**Results:**

A total of 57 students consented to participate, of which 55 completed both the pre- and post-assessments (3.5% attrition). The mean age of the participants was 24.8 ± 1.9 years, 50.9% (*n* = 29) identified as women, and 68.4% (*n* = 39) identified as white. Pre-intervention, 42.1% (*n* = 24) of students met criteria for smartphone addiction and 77.2% (*n* = 44) met criteria for poor sleep quality. In addition, 22.8% (*n* = 13) of participants had high emotional exhaustion, 64.9% (*n* = 31) high depersonalization, and 42.1% (*n* = 24) low sense of personal accomplishment prior to the intervention. Following the two-week intervention, participants showed improvements in emotional exhaustion (*p* = 0.001, Cohen’s d = 0.353), depersonalization (p = 0.001, Cohen’s d = 0.411) low sense of personal accomplishment (*p* = 0.023, Cohen’s d = 0.275), perceived stress (*p* < .001, Cohen’s d = .334), and sleep quality (*p* < 0.001, Cohen’s d = 0.925). The number of participants who reported poor sleep quality decreased to 41.8% (*n* = 23), demonstrating a significant decline (*p* = 0.026). Participants also improved subjective sleep quality (*p* < 0.001, Cohen’s d = 1.033), sleep duration (*p* = 0.001, Cohen’s d = 0.431), sleep latency (p < 0.001, Cohen’s d = 0.433), and sleep efficiency (*p* = 0.021, Cohen’s d = 0.673).

**Conclusions:**

These findings suggest that the two-week sunrise alarm clock protocol with electronic device removal was effective in improving sleep quality and reducing burnout scores, and perceived stress. However, additional research comparing this intervention to a proper control group is needed to draw meaningful conclusions about the effectiveness of this intervention.

Burnout is a psychological syndrome that occurs in response to chronic interpersonal stressors on the job [1]. Maslach and colleagues define burnout as a combination of three dimensions, emotional exhaustion (e.g., overwhelming exhaustion), depersonalization (e.g., cynicism, detachment), and low sense of personal accomplishment (e.g., ineffectiveness) [2, 3]. Several large international studies show physicians, residents, and medical students report higher rates of burnout compared to other occupations [4–9]. The effects of burnout have serious consequences on patient safety, quality of care, professionalism, and patient satisfaction [10–12]. Moreover, burnout is associated with numerous negative health outcomes, including coronary heart disease, musculoskeletal pain, type 2 diabetes, mental health issues, suicidal ideation, substance abuse, and sleep disorders [13–16].

While sleep disorders are critically linked to burnout [17, 18], poor sleep quality itself is associated with negative health outcomes, including depression, anxiety, alcohol abuse, and thoughts of self-harm [19–21]. Sleep is restorative for daily functioning [22]. Both the quality and quantity of sleep affect an individual’s ability to cope with emotional challenges, particularly emotionally painful events [22]. Conversely, sleep deprivation and sleep disturbances trigger negative emotional reactivity and diminish the effect of positive emotions [22]. For these reasons, practices and behaviors that promote consistent and uninterrupted sleep are important. Sleep hygiene involves a set of practices and behaviors including adequate exposure to sunlight, regular exercise, limiting daytime naps to 30 min, consistency with a set sleep schedule, avoidance of stimulants (e.g., caffeine, nicotine) and alcohol close to bedtime, refraining from eating large meals late in the evening, and limited exposure to smartphones and computer screens at bedtime [23]. These practices and behaviors have been shown to improve sleep quality and increase daytime alertness [23].

Several studies have demonstrated the deleterious effects of smartphone use on sleep [24]. Exposure to smartphone light-emitting diode (LED) light at night may affect circadian rhythms, melatonin secretion, and alteration of mood and cognition [24]. Smartphone overuse is associated with other negative health consequences, such as increased anxiety, impulsive behaviors, behavioral addiction, and alcohol use disorder [25, 26]. Problematic smartphone use [27], sometimes classified as an addiction [28], is characterized by smartphone use with negative consequences, such as detrimental effects on attention, financial problems, dangerous use, and problematic social and academic behaviors [25]. Problematic smartphone use has been compared to Internet addiction, and may contribute to poor academic or professional achievement [29].

Interestingly, smartphone overuse is associated with higher burnout scores, in particular emotional exhaustion and depersonalization, in medical students [30]. This may be particularly relevant considering the percentages of medical students exhibiting smartphone addiction across the world (29.8% in Anhui Province, China [31]; 39.9% in Delhi, India [28], 36.5% in Jeddah, Saudi Arabia [32], 22.3% in Ohio, United States [30]). Medical students experience disproportionately higher burnout rates compared to similarly aged college graduates [4]. In fact, a recent systematic review and meta-analysis found 44.2% of medical students experienced burnout, with estimates of 40.8% for emotional exhaustion, 35.1% for depersonalization, and 27.4% for low sense of personal accomplishment [33]. Moreover, medical students have a higher prevalence of poor sleep compared to the general population [34]. Poor sleep quality and sleep deprivation are critically linked to higher burnout scores in medical students [19, 20]. Thus, successful interventions that address burnout, sleep, and smartphone use are needed for medical students.

One simple behavioral approach to improve sleep quality and reduce burnout is limiting exposure to smartphones and other electronic devices at bedtime and replacing them with gentle wake-up alarms. Providing an alternative alarm clock is critical given most Americans use the alarm clock feature on their smartphone devices to wake up each morning [35]. Sunrise alarm clocks may be a viable behavioral intervention to improve sleep quality because of their reliance on dawn simulation technology. Studies have shown that dawn stimulation in a small cohort of participants found modest improvements in subjective quality of sleep [36] and light exposure during the last 30 min of habitual sleep increased subjective alertness [37]. No known research has addressed the efficacy of gentle wake-up methods with medical students. Thus, the purpose of this study was twofold: 1) to assess the feasibility of implementing a sunrise (light-controlled) alarm clock intervention with medical students in combination with electronic device removal at bedtime and 2) to evaluate the impact of the intervention on burnout, perceived stress, and sleep quality. We hypothesized that the intervention would improve medical students’ sleep quality and decrease their perceived stress and burnout, specifically emotional exhaustion, depersonalization, and low sense of personal accomplishment.

## Methods

This feasibility study evaluated the efficacy of a sunrise alarm clock intervention in combination with electronic device removal at bedtime. The study measured indicators of medical students’ emotional well-being before and after a two-week protocol in order to assess changes in burnout, perceived stress, and sleep quality pre- and post-intervention. The University Office of Research Compliance approved the protocol (18-X-157) and all recruitment procedures and materials.

### Participants

First- and second-year medical students enrolled at a large medical school in the Midwest were invited to participate in a two-week intervention. Students were recruited via email using class mailing lists. The research team distributed a recruitment email on April 23, 2018 and a reminder email one week later. Interested students were instructed to schedule an in-person visit to review the study protocol, provide informed consent, and complete the pre-assessment. Participants were recruited on a rolling basis. The first participant completed their pre-assessment on April 23, 2018 and the last participant completed their post-assessment on May 28, 2018. Participants received a $10 gift card for completion of each assessment. In addition, participants kept the alarm clock from the study. Participation in the study was voluntary.

### Intervention protocol

For the two-week protocol, all participants were provided a sunrise alarm clock and instructed to use it for their regular sleep-wake cycles instead of their current alarm clock (e.g., smartphone, tablet, computer). Next, participants were asked to turn off their smartphone and all other electronic devices at night for the entire two-week protocol. Night was defined as when the participant chose to go to bed. Below is a bulleted list of the protocol participants were asked to complete for this protocol:

1. Replace current alarm clock with the sunrise alarm clock received from the study.
2. Turn off smartphone and all electronic devices at bedtime.
3. Follow this protocol for two weeks.

Participants were not asked to change any of their other behaviors or habits in this protocol.

### Measures

Participants completed a brief sociodemographic (age, sex, race, year in medical school) and health factors (height, weight, BMI) questionnaire. Note, the data collected on race followed the United States Census categories (i.e., White, Hispanic/Latino/Spanish, Black/African American, Asian, American Indian/Alaskan Native, Middle Eastern or North African, Native Hawaiian/Pacific Islander, and Other) [38]. In addition, participants completed the free response questions about alarm clock use over the two-week period. Specifically, participants were asked the number of days they used the provided alarm clock to wake up and the number of nights they had the smartphone and other electronic devices turned off and out of their bedrooms. These free response questions were used to measure adherence to the protocol. Also, participants completed the following validated measures:

#### Maslach burnout inventory-human services survey (MBI-HSS) [39]

A 22-item measure assessing Emotional Exhaustion (e.g., “I feel emotionally drained by my work”), Depersonalization (e.g., “I feel tired when I get up in the morning and have to face another day at work”), and Low Sense of Personal Achievement (e.g., “I accomplish many worthwhile things in this job.”). Participants scored each item on a seven-point scale, where 0 was “never,” 1 was “sometimes per year or less often,” 2 was “once a month or less often,” 3 was “several times a month,” 4 was “once a week,” 5 was “several times a week,” and 6 was “daily.” Consistent with prior research, high emotional exhaustion scores were ≥ 27, high depersonalization scores were ≥ 10, and low sense of personal accomplishment scores ≤33 [40, 41]. The MBI-HHS was administered at pre- and post-intervention.

#### Perceived stress scale (PSS-4) [42]

A 4-item measure assessing psychological stress over the past month. This brief self-assessment measures the degree to which an individual perceives their lives to be stressful (e.g., “In the last month, how often have you felt that you were unable to control the important things in your life?”). Participants scored each item on a four-point scale, where 0 was “never,” 1 was “almost never,” 2 was “sometimes,” 3 was “fairly often,” and 4 was “very often.” Scores ranged from 0 to 16, with higher scores indicating a higher perception of stress. Prior studies have demonstrated internal consistency for the PSS-4 to be between 0.67–0.73 [42–44]. The PSS-4 was administered at pre- and post-intervention.

#### Pittsburgh sleep quality assessment (PSQI) [45]

A 19-item self-reported measure assessing quality and patterns of sleep in adults (e.g., “During the past month, how often have you had trouble sleeping because you cannot get to sleep within 30 minutes?” and “During the past month, how often have you taken medicine to help you sleep?”). Participants provided qualitative and quantitative data describing usual sleep habits during the past month only. The scale was then scored to create a seven-component measurement, with a total score of “5 or greater” indicating poor sleep quality. In addition, individual components were calculated for pre-post analysis, including subjective sleep quality, sleep duration, sleep latency, and sleep efficiency. Subjective sleep quality was a rating of the participants’ sleep quality in the past month, ranging from 0 to 3 with ‘0 = very good’ and ‘3 = very bad’. Sleep duration was the number of hours of actual sleep participants got at night. Sleep latency was the number of minutes it took to fall asleep each night. Lastly, sleep efficiency was calculated by dividing the sleep duration by total number of hours in bed multiplied by 100. The PSQI was administered at pre- and post-intervention.

#### Smartphone addiction scale short version (SAS-SV) [46]

A 10-item measure assessing smartphone addiction (e.g., “Using my smartphone longer than I had intended” and “Won’t be able to stand not having a smartphone.”) Participants scored items on a six-point scale, where 1 was “strongly disagree,” 2 was “disagree,” 3 was “weakly disagree,” 4 was “weakly agree,” 5 was “agree,” and 6 was “strongly agree.” Internal consistency of the measure is excellent with a Cronbach’s alpha correlation coefficient of 0.91 [46]. The cutoff value for smartphone addiction is 31 in male participants and 33 in female participants [46]. The SAS-SV was administered at the pre-intervention only.

### Data collection

Participants completed the pre- and post-assessments via the online questionnaire service Qualtrics (Provo, UT: Qualtrics). Participants attended one on-campus visit in a confidential space to provide written informed consent and complete the pre-assessment. At the visit, participants were asked to read and sign an informed consent form prior to participation. The informed consent document explicitly informed potential participants that their responses had no bearing on academic performance, and that they could withdraw consent at any time during the study. If they consented to participate in the study, they were assigned a unique identification number and asked to complete the pre-assessment measures via the Qualtrics link on a password-protected desktop computer in the confidential space. After participants completed the two-week intervention protocol, one member of the research team distributed a Qualtrics link for the post-assessment. To link pre- and post-assessment responses, participants were instructed to enter their unique identifier for both the pre- and post-assessment via Qualtrics. Completion of each assessment lasted approximately 15 min.

### Data analysis

Basic sociodemographic characteristics of participants were assessed using descriptive statistics. Frequencies of individual question responses were also calculated. Chi-square tests, Fisher’s exact tests, independent samples t-tests, one-way analysis of variance (ANOVA), and Pearson’s correlation coefficients were used to examine differences in emotional exhaustion, depersonalization, low sense of personal accomplishment, perceived stress, sleep quality, and smartphone addiction by demographic characteristics. Specifically, we examined differences by age, gender, race, year in medical school, and BMI based on prior literature in the US that showed older age was protective from higher burnout scores, women were more likely to screen positive for depression and fatigue compared to men, non-White medical students had lower mental health scores compared to White students, burnout scores increase after year-one and year-three of medical school, and poorer sleep quality is associated with higher BMI [4, 47–49]. We dichotomized the race variable by participants who self-identified as White (0) and students who self-identified as non-White [1]. Next, the research team performed Chi-square tests or Fisher’s exact tests and paired t-tests to examine changes in emotional exhaustion, depersonalization, low sense of personal accomplishment, perceived stress, and sleep quality before and after the intervention. In addition, the research team determined effect sizes using Cohen’s d by calculating the mean difference between the pre- and post-assessment total scores divided by the pooled standard deviation. Finally, we assessed adherence to the intervention via the two free response questions using descriptive statistics and calculated change scores for emotional exhaustion, depersonalization, low sense of personal accomplishment, perceived stress, and sleep quality. Then, we conducted Pearson’s correlation coefficients with the change scores and the number of days the participants adhered to the protocol to determine whether greater adherence was correlated with a greater change in outcomes. We defined statistical significance as a *p*-value less than 0.05, and conducted analyses in SPSS statistical software version 26.0 (Chicago, IL: SPSS Inc.)

## Results

Of the 286 osteopathic medical students enrolled at the main campus, 57 consented to participate in the feasibility study. Two participants did not complete the post-assessment (3.5% attrition). Thus, a total of 55 participants completed both the pre- and post-assessments. The mean age of the participants was 24.8 ± 1.9 years, 50.9% (*n* = 29) identified as women, 68.4% (*n* = 39) identified as white, and 66.7% (*n* = 38) were second-year medical students. On average, participants were 67.7 ± 4.9 in. tall and weighed 167.4 ± 36.0 pounds. The mean body mass index (BMI, kg/m^2^) was 25.8 ± 5.0. Additional demographic and health characteristics are presented in Table 1. Overall, the demographic data in this study were similar to findings from a larger research study with the same first- and second-year students (age = 24.9 ± 2.3, t = .3065 *p* = .759; 52.3% women (*n* = 139), χ^2^ = .0467, *p* = 0.829; 73.3% White (*n* = 195), χ^2^ = .630, *p* = 0.529; BMI = 24.9 ± 4.5, .630, p = 0.529) on burnout, sleep quality, and smartphone addiction [30] as well as the general population of first- and second-year students (*n* = 495; age = 25.0 ± 2.2; 52.7% women (*n* = 257); 76% White (*n* = 376); BMI = unknown) from this institution.

**Table 1:** Participants’ Demographic and Health Characteristics (*n* = 57)

Variable	All participants n (%)
Age (years)	24.8 ± 1.9
Gender	
Women	29 (50.9)
Men	28 (49.1)
Race	
American Indian/Alaskan Native	0 (0)
Asian	8 (14.0)
Black/African American	3 (5.3)
Hispanic/Latino/Spanish	0 (0)
Middle Eastern/North African	3 (5.3)
Mixed Race	4 (7.0)
Native Hawaiian/Pacific Islander	0 (0)
White	39 (68.4)
Year in osteopathic medical school	
OMS I	19 (33.3)
OMS II	38 (66.7)
Height (inches)	67.7 ± 4.9
Weight (pounds)	167.4 ± 36.0
Body Mass Index (BMI; kg/m^2^)	25.8 ± 5.0
Smartphone Addiction	24 (42.1)

Values missing for Height (*n* = 1), BMI (*n* = 1)

### Burnout

Prior to the intervention, 22.8% (*n* = 13) of participants reported high emotional exhaustion, 64.9% (n = 37) high depersonalization, and 42.1% (*n* = 24) low sense of personal accomplishment. Only 14.0% (*n* = 8) participants reported high burnout scores in all three dimensions. Dimensions of burnout did not differ by age (emotional exhaustion: t = 0.931, *p* = 0.356; depersonalization: t = 0.538, *p* = 0.593; low sense of personal accomplishment: t = − 0.267, *p* = 0.791), gender (emotional exhaustion: χ^2^ = 2.270, *p* = 0.132; depersonalization: χ^2^ = 0.210, *p* = 0.647; low sense of personal accomplishment: χ^2^ = 0.013, *p* = 0.910), race (emotional exhaustion: χ^2^ = 1.892, *p* = 0.756; depersonalization: χ^2^ = 2.569; *p* = 0.632; low sense of personal accomplishment: χ^2^ = 3.947, *p* = 0.413), year in medical school (emotional exhaustion: χ^2^ = 0.050, *p* = 0.823; depersonalization: χ^2^ = 0.616, *p* = 0.432; low sense of personal accomplishment: χ^2^ = 2.915, *p* = 0.088), or BMI (emotional exhaustion: t = − 0.429, *p* = 0.670, depersonalization: t = 1.994, *p =* 0.051; low sense of personal accomplishment: t = 1.980, *p* = 0.053). Interestingly, participants who met criteria for smartphone addiction were more likely to report high depersonalization (χ^2^ = 6.176, *p* = 0.013), but not high emotional exhaustion (χ^2^ = 0.113, *p* = 0.736) or low sense of personal accomplishment (χ^2^ = 1.060, *p* = 0.303). High emotional exhaustion (t = 2.546, *p* = 0.014), high depersonalization (t = 4.154, *p* < 0.001), and low sense of personal accomplishment (t = 2.707, *p* = 0.009) were associated with poorer sleep quality.

Burnout scores improved post-intervention in all three dimensions (see Table 2). Participants reported positive improvements in emotional exhaustion (mean improvement = 3.0 ± − 6.4, *p* = 0.001, Cohen’s d = 0.353; see Table 2), depersonalization (mean improvement = 2.7 ± − 5.6, p = 0.001, Cohen’s d = 0.411) and low sense of personal accomplishment (mean improvement = − 1.8 ± 5.8, *p* = 0.023, Cohen’s d = 0.275). The effect size for all three improvements ranged between 0.275 and 0.411, indicating small to medium effects. In addition, the number of participants with high levels of emotional exhaustion (χ^2^ = 5.867, *p* = 0.015), high depersonalization (χ^2^ = 13.133, *p* < 0.001), and low sense of personal accomplishment (χ^2^ = 13.750, p < 0.001) decreased from pre- to post-intervention. In addition, only 5.3% (*n* = 3) participants reported high burnout scores in all three dimensions post-intervention (χ^2^ = 17.610, *p* = 0.040). Poorer sleep quality remained associated with high emotional exhaustion (t = 3.583, *p* = 0.001) and high depersonalization (t = 2.738, *p* = 0.008), but not low sense of personal accomplishment t = 1.338, *p* = 0.187) post-intervention.

**Table 2:** Pre- and Post-Assessment of Perceived Stress, Emotional Exhaustion, Depersonalization, Low Sense of Personal Achievement, and Sleep Quality (*n* = 55)

s	Pre-Intervention	Post-Intervention	Mean change	*P*	Cohen’s *d*
Perceived Stress	6.3 ± 3.1	5.3 ± 3.5	1.0 ± 2.7	.012	.334
Emotional Exhaustion	19.2 ± 8.3	16.2 ± 9.2	3.0 ± 6.4	.001	.353
Depersonalization	12.5 ± 7.5	9.9 ± 6.1	2.7 ± 5.6	.001	.411
Personal Achievement	34.9 ± 8.0	36.7 ± 7.3	−1.8 ± 5.8	.023	.275
Global Sleep Quality Score^a^	7.3 ± 3.2	4.7 ± 2.5	2.6 ± 2.7	<.001	.925
Subjective Sleep Quality Score^b^	1.4 ± .7	.7 ± .7	.7 ± .7	<.001	1.033
Sleep Duration (hours)^c^	6.7 ± .9	7.1 ± .8	.4 ± .8	.001	.431
Sleep Latency (minutes)^d^	43.5 ± 64.5	22.1 ± 18.6	21.4 ± 62.7	<.001	.433
Sleep Efficiency (%)^e^	84.9 ± 10.2	91.0 ± 7.7	5.7 ± 8.6	.021	.673

^a^ A total score of “5” or greater is indicative of poor sleep quality

^b^ Subjective Sleep Quality is a rating of sleep quality in the past month, ranging from 0 to 3 with ‘0 = very good’ and ‘3 = very bad’

^c^Sleep duration is the number of hours of actual sleep a person gets at night

^d^Sleep latency is the number of minutes it takes to fall asleep each night

^e^Sleep efficiency is equal to (total number of hours of asleep)/(total number of hours in bed)*100

### Perceived stress

Pre-intervention, participants reported total perceived stress scores of 6.3 ± 3.1 (see Table 2). Perceived stress did not differ by age (r = 0.184, *p* = 0.171), gender (t = − 1.626, *p* = 0.110), race (F = 0.583, *p* = 0.898), year in medical school (t = − 1.118, *p* = 0.258), or BMI (r = 0.089, *p* = 0.516). Higher scores of perceived stress were associated with high emotional exhaustion (t = 6.041, *p* < 0.001), high depersonalization (t = 4.159, p < 0.001), and low sense of personal accomplishment (t = 3.653, *p* = 0.001). Similarly, higher perceived stress scores were correlated with poorer sleep quality (r = 0.585, *p* < 0.001). Higher perceived stress scores were not associated with smartphone addiction (t = 1.209, *p* = 0.232).

Perceived stress scores improved post-intervention (mean improvement = 1.0 ± − 2.74, p < 0.001, Cohen’s d = 0.334; see Table 2). Post-intervention, higher scores of perceived stress remained associated with high emotional exhaustion (t = 3.600, *p* = 0.001), high depersonalization (t = 2.297, *p* = 0.026), and low sense of personal accomplishment (t = 2.324, *p* = 0.024). In addition, higher perceived stress scores were correlated with poorer sleep quality (r = 0.337, *p* = 0.012).

### Sleep quality

Pre-intervention, 77.2% (*n* = 44) of participants reported total global sleep quality scores ≥5, indicating poor sleep quality, with mean scores of 7.3 ± 3.2. Participants rated their subjective sleep quality between fairly good and fairly bad (mean = 1.4 ± 0.7). On average, they slept 6.7 ± 0.9 h per night, taking an average of 43.5 ± 64.5 min to fall asleep, generating a sleep efficiency score of 84.9 ± 10.2%. Sleep quality did not differ by age (t = 0.689, *p* = 0.494), gender (t = − 1.182, *p* = 0.242), race (F = 0.606, *p* = 0.660), year in medical school (t = 0.352, *p* = 0.726), or BMI (t = − 0.040, *p* = 0.968). As stated above, sleep quality was associated with high emotional exhaustion, high depersonalization, low sense of personal accomplishment, higher perceived stress scores, and smartphone addiction (t = 2.164, *p* = 0.035).

Global sleep quality scores improved post-intervention (mean improvement = 2.6 ± 2.7, *p* < 0.001, Cohen’s d = 0.925; see Table 2). The Cohen’s d of 0.925 represents a very large effect in improved global sleep quality. Further, a total of 41.8% (*n* = 23) participants reported total global sleep quality scores ≥5, showing a significant drop pre- and post-intervention (*p* = 0.026). Participants also improved subjective sleep quality (mean improvement = 0.7 ± 0.7, *p* < 0.001, Cohen’s d = 1.033), sleep duration (mean improvement = 0.4 ± 0.8, *p* = 0.001, Cohen’s d = 0.431), sleep latency (mean improvement = 21.4 ± 62.7, p < 0.001, Cohen’s d = 0.433), and sleep efficiency (mean improvement = 5.7 ± 8.6, *p* = 0.021, Cohen’s d = 0.673). The largest magnitude of change occurred with the subjective sleep quality, with a Cohen’s d of 1.033, indicating another very large effect. As stated above, poorer sleep quality was associated with high emotional exhaustion and high depersonalization, but not low sense of personal accomplishment post-intervention. Also, higher perceived stress scores remained correlated with poorer sleep quality post-intervention. Finally, pre-intervention smartphone addiction was no longer associated with poorer sleep quality post-intervention (χ^2^ = 0.399, *p* = 0.692).

### Smartphone addiction

Pre-intervention, 42.1% (*n* = 24) of participants met criteria for smartphone addiction. Smartphone addiction did not differ by sex (χ^2^ = .013, *p* = 0.910), race (χ^2^ = 3.947, *p* = 0.413), year in medical school (χ^2^ = 1.295, *p* = 0.255), or BMI (t = 1.930, *p* = 0.059). However, participants who were older were more likely to meet criteria for smartphone addiction (t = 2.144, *p* = 0.036). Smartphone addiction was not associated with higher perceived stress scores (t = 1.209, *p* = 0.232). On the other hand, smartphone addiction was associated with high depersonalization (χ^2^ = 6.176, *p* = 0.013), but not emotional exhaustion (χ^2^ = 0.113, *p* = 0.736) or low sense of personal accomplishment (χ^2^ = 1.060, *p* = 0.303) pre-intervention. Further, participants with smartphone addiction were more like to have poorer sleep quality (χ^2^ = 2.164, *p* = 0.035) before the intervention.

Post-intervention, pre-intervention smartphone addiction was not associated with high emotional exhaustion (χ^2^ = 0.437, *p* = 0.509), high depersonalization (χ^2^ = 0.596, *p* = 0.440), or low sense of personal accomplishment (χ^2^ = 0.382, *p* = 0.537). Further, pre-intervention smartphone addiction was not associated with higher perceived stress scores (t = 0.126, *p* = 0.900) or poorer sleep quality (t-0.399, *p* = 0.692).

### Intervention adherence

On average, participants used the sunrise alarm clock 11.4 ± 3.4 days out of 14 days in the two-week period and turned off or removed their smartphones and other electronic devices 11.3 ± 3.4 days. Neither adherence to the sunrise alarm clock nor adherence to the smartphone protocol were correlated with the change scores for perceived stress (r = 0.003, *p* = 0.983; r = 0.027, *p* = 0.845, respectively), emotional exhaustion (r = − 0.047, *p* = 0.740; r = 0.079, *p* = 0.569), depersonalization (r = 0.079, *p* = 0.580; r = 0.133, *p* = 0.337), low sense of personal accomplishment (r = 0.087, *p* = 0.540; r = − 0.027, *p* = 0.849), or sleep quality (r = − 0.183, *p* = 0.195; r = 0.128, *p* = 0.358).

## Discussion

In this feasibility study, we evaluated the impact of a sunrise alarm clock intervention with electronic device removal at bedtime on medical student well-being and sleep quality. Prior to the intervention, 22.8% of participants reported high emotional exhaustion, 64.9% high depersonalization, 42.1% low sense of personal accomplishment, 77.2% met criteria for poor sleep quality, and 42.1% participants met criteria for smartphone addiction. Among the participants, poorer sleep quality was associated with high emotional exhaustion, high depersonalization, and low sense of personal accomplishment, higher perceived stress scores, and smartphone addiction. Following the intervention, we observed improvements in emotional exhaustion, depersonalization, low sense of personal accomplishment, perceived stress, and sleep quality. Specifically, participants reported improvements in global sleep quality, subjective sleep quality, sleep duration, sleep latency, and sleep efficiency. The largest magnitude of change occurred with subjective sleep followed by global sleep quality, both indicating very large effects. At post-intervention, only two-fifths (41.8%) of participants met criteria for poor sleep quality, which was no longer associated with pre-intervention smartphone addiction. These findings suggest that the two-week sunrise alarm clock protocol with electronic device removal at bedtime was effective in improving sleep quality and reducing perceived stress and burnout in medical students. However, additional research comparing this intervention to a proper control group is needed to draw meaningful conclusions about the effectiveness of this intervention.

The behavioral impacts of problematic smartphone use have been well-documented. Smartphones can be transferred easily between home and work environments. This boundary dissolution may create stress and anxiety for some people [25, 50]. For the students in this study, boundary dissolution between school and home life may have been exemplified by the high scores of emotional exhaustion, depersonalization, low sense of personal accomplishment, and perceived stress pre-intervention. A study by Thomee and colleagues [51] found young adult smartphone users felt that they were expected to be reachable at all times of day, though most of them did not consider increased accessibility to be stressful [51]. Similarly, the students in this study may have felt the need to be reached at all times of day. Future studies should measure medical students’ perceived need for accessibility. Additionally, the increase in reward-related responses to smartphone use may reinforce excessive reassurance-seeking [25], as well as poor self-esteem and insecure attachments [25]; however, these factors have not been firmly established. Future research may expand on behavioral responses, as well as the effects of smartphone addiction and associated sleep deprivation on emotional experiences such as anger, fear, or happiness.

In this study, smartphone addiction was associated with high depersonalization and poorer sleep quality prior to the intervention. The negative effects of nighttime media use are partly biological in nature. The human eye is most sensitive to blue light [52], which is emitted in proportionately higher quantities from LED displays when compared to incandescent or ambient light. Blue light strongly stimulates several areas of the brain that are responsible for circadian rhythm and mood-related effects [53]. For these reasons, blue light is thought to evoke the most potent effects on stress and mood when compared with other colors of light [24].

Mistimed light exposure may have other deleterious outcomes. While a direct link to smartphone use remains uncertain, current theories include modulation of the hypothalamic-pituitary-adrenal (HPA) axis, downregulation of melatonin, and upregulation of stress-related hormones such as glucocorticoids [53]. Functional MRI has allowed researchers to explore the neurobiological response to smartphone use. Areas of the brain that are responsible for affective signals of reward, including interconnectivity between the nucleus accumbens and orbitofrontal cortex, have been associated with problematic smartphone use [54]. In addition to these neurobiological effects, nighttime smartphone use may underlie sleep loss, which is an important predictor of mood disorders such as anxiety and depression, as well as general cognitive effects [55].

### Limitations

Limitations of this study include data from one medical school, selection bias, self-reported data, confounding bias, and lack of a control group. Data from one medical school limits the generalizability of findings to other programs. Only students who were enrolled in pre-clinical education (year 1 and year 2) were included in the study; medical students in clinical education (year 3 and year 4) did not have the opportunity to participate. Third- and fourth-year students were excluded because they were not physically present on the medical school campus; rather, they were completing rotations at different clinical sites across the state. Future research should include medical students of all four years. Next, our findings may be susceptible to selection bias, as students who volunteered to participate may have been more willing or motivated to participate in an intervention designed to improve sleep and reduce burnout. In addition, the self-reported data collected via the pre- and post-assessments may be vulnerable to social desirability bias [56]. To minimize bias, the researchers informed participants that their responses were confidential and all of the information provided by them was labeled with an identification number, which they received during their one on-campus visit, and not their name. Further, the investigators emphasized the voluntary nature of participation and explicitly informed the participants that their responses had no bearing on their academic performance. Importantly, the intervention demonstrated reductions in symptoms of emotional exhaustion, depersonalization, and low sense of personal accomplishment; however, pre-intervention, only 14.0% of participants met criteria for high burnout scores in all three dimensions. Thus, the overwhelming majority of participants experienced symptoms of burnout in a combination of different dimensions. Post-intervention, 5.3% of participants met criteria for high burnout scores in all three dimensions. Findings must be considering in the context of all three dimensions of burnout.

In addition, the inclusion of the sunrise alarm clock introduced confounding bias to the study because the study design did not allow us to determine whether the improvements in sleep quality, perceived stress, and burnout were attributed to the removal of the smartphones at bedtime, the sunrise alarm clock, a combination of the two, or other factors. Finally, this feasibility study did not include a control group, which limits our ability to conclude whether the changes observed in the study were due to the intervention or other factors. Thus, future research must include a control group in order to draw meaningful conclusions. We plan to conduct a randomized-control trial removing smart phones and all electronic devices at bedtime and replacing those devices with a sunrise alarm clock or standard alarm clock. The aim will be to compare the effectiveness of this sunrise alarm clock intervention versus a standard alarm clock versus a control group (i.e., wait list control group). This three-arm parallel randomized controlled trial will determine whether the sunrise alarm clock intervention is an effective intervention and whether the sunrise alarm clock is more effective than a standard alarm clock.

## Conclusions

These findings suggest that the two-week sunrise alarm clock protocol with electronic device removal was effective in improving sleep quality and reducing perceived stress and burnout. However, additional research comparing this intervention to a proper control group is needed to draw meaningful conclusions about the effectiveness of this intervention. If these findings are confirmed via a randomized controlled trial, this intervention has the potential to improve medical students’ health and well-being.

## Data Availability

The dataset generated and analyzed for the current study are not available for public use; however, they are available upon request from the corresponding author.

## Abbreviations

LED: Light-emitting diode
HPA: Hypothalamic-pituitary-adrenal
ANOVA: Analysis of variance
